# Lipopolysaccharide Exposure during Pregnancy Leads to Aortic Dysfunction in Offspring Rats

**DOI:** 10.1371/journal.pone.0102273

**Published:** 2014-07-15

**Authors:** Shanyu Zhao, Haigang Zhang, Dayan Cao, Ya Liu, Xiaohui Li

**Affiliations:** 1 Institute of Materia Medica and Department of Pharmaceutics, College of Pharmacy, Third Military Medical University, Chongqing, PR China; 2 Department of Pharmacology, College of Pharmacy, Third Military Medical University, Chongqing, PR China; Max-Delbrück Center for Molecular Medicine (MDC), Germany

## Abstract

**Background:**

Prenatal exposure to Lipopolysaccharide (LPS) produces hypertension in adult offspring rats. The present study was to explore the effects of prenatal inflammation on morphological and functional changes in the aorta from offspring rats and to further assess its susceptibility to cardiovascular diseases.

**Methods and Results:**

Pregnant rats were treated intraperitoneally on gestation Days 8, 10 and 12 with saline, LPS (0.79 mg/kg), or pyrrolidine dithiocarbamate (PDTC, 100 mg/kg)+LPS, respectively. Aortic ring reactivity and histopathological alteration were analyzed in offspring at the age of 12 weeks. The detections of connexin (Cx) 37, Cx40, Cx43, and Cx45, including immunofluorescent patterns, protein levels and mRNA expression in the aorta, were performed as well. Furthermore, the expressions of Nuclear factor (NF)-κB (p65), IκBα, phospho-IκBα and IκBβ were determined. The results showed that prenatal LPS exposure leads to morphological abnormalities and impaired aortic reactivity in offspring. Prenatal LPS exposure also decreased the protein and mRNA expression of Cx37 in the aorta from offspring rats. NF-κB and phospho-IκBα levels were both increased, IκBα level, however, was decreased in the aorta of offspring from the maternal LPS exposure compared to the controls. Simultaneously, PDTC treatment markedly reversed the action of LPS.

**Conclusions:**

Decreased expression of Cx37 contributed to the aortic dysfunction of prenatal LPS exposure offspring, which should be associated with NF-κB activation.

## Introduction

Cardiovascular disease remains the principle cause of morbidity and mortality in western countries as well as in the developing world. Risk factors such as genetics and environmental factors are thought to play a role in these diseases but does not account for all cases [1]. Epidemiological studies have indicated that a suboptimal intrauterine environment, resulting in impaired fetal growth, is associated with an increased risk of the incidence of cardiovascular-related complications [2], [3]. Evidence from various animal experiments revealed that alterations in nutrition and endocrine status during pregnancy can predispose adult offspring to increased blood pressure, dysglycemia, dyslipidemia, and obesity, which correspond to cardiovascular disease risk factors concomitant with individual genetic and environmental backgrounds [4]–[7]. Recently, we reported a rat model of maternal non-bacterial inflammatory response induced by lipopolysaccharide (LPS) or zymosan during early and mid- pregnancy where the offspring developed elevated blood pressure as early as 6 wk of age, which progressively increased with age. In addition, the offspring showed significantly increased body weight and abdominal fat weight [8], [9]. Furthermore, alterations in the local renin-angiotensin system and renal damage resulting from prenatal LPS or Zymosan exposure were interpreted to the elevated blood pressure in adult offspring rats [10], [11]. However, the mechanisms behind relationship still remain to be elucidated.

Cardiovascular homeostasis relies on the integrity of the vascular endothelium, and the healthy endothelium not only modulates the tone of the underlying vascular smooth muscle cells (SMCs) but also inhibits SMCs proliferation and migration, monocyte and platelet adhesion, and the synthesis of inflammatory cytokines, thereby exhibiting important pathophysiological roles. Maternal inflammation and the subsequent inflammatory responses could contribute to a suboptimal intrauterine environment [12]. Fetal adaptations to the adverse intrauterine environment may include altered cellular differentiation and tissue growth to ensure short-term survival but may also result in impaired cardiovascular structure and function in offspring [13], [14]. Considerable numbers of studies have demonstrated that maternal hypercholesterolemia, hypoxia during pregnancy, or fetal exposure to elevated glucocorticoid levels lead to vascular dysfunction in adult offspring [15]–[17]. Moreover, the importance of preexisting vascular abnormalities in the development of cardiovascular complications has been revealed [18], [19]. To date, however, the specific effects of maternal inflammation exposure on the function and structure of the vasculature in adult offspring remain to be discussed.

In the present study, the rat model of maternal LPS exposure during pregnancy was duplicated to address the impact of prenatal inflammation on morphological and functions changes in the aorta in 12-wk-old offspring and to further assess their susceptibility to cardiovascular-related complications.

## Methods

### Animals

Nulliparous pregnant time-mated Sprague–Dawley rats were obtained from the Experimental Animal Center of the Third Military Medical University (Chongqing, China). All animals had free access to standard laboratory rat chow and tap water. Until parturition, rats were housed individually in a room at constant temperature (24°C) and under a 12 h light–dark cycle. The pups were raised with a lactating mother until 4 weeks of age, at which time they were removed to cages containing three or four other rat pups. The present study conforms to the *Guide for the Care and Use of Laboratory Animals* published by the US National Institutes of Health (NIH Publication No. 85–23, revised 1996; http://www.nap.edu/readingroom/books/labrats/index.html). The study protocol was approved by the Ethical Committee for Animal Experimentation of the Third Military Medical University.

After 5 days of acclimation, the pregnant rats were randomly divided into 3 groups of 8 animals each: (i) a control group, administered 0.5 ml i.p. saline on gestation Days 8, 10 and 12, (ii) an LPS group, given 0.79 mg/kg i.p. LPS (Sigma Chemical, St. Louis, MO, USA) on gestation Days 8, 10 and 12, and (iii) a pyrrolidine dithiocarbamate (PDTC) + LPS treated group, given 100 mg/kg i.p. PDTC 1 h before LPS. After birth, the litters were counted and weighed. The litter size was then reduced to eight pups to ensure equal nutrient access for all of the offspring. Neonatal rats were cared for by their mothers until they were weaned, after which they were fed standard rat chow. At that time, all offspring rats from each group of dams were mixed together again and randomly allocated to cages. Drinking water and food were available *ad libitum* throughout the study. At 12 weeks of age, offspring contained males and females from each group were selected randomly and anesthetized with sodium pentobarbital (50 mg/kg) administered intraperitoneally. The aorta from the aortic arch to the thoracic aorta was gently removed for subsequent analysis.

### Aortic rings reactivity

#### Preparation of rat aortic rings

The removed-thoracic aorta were stored in a Krebs- Henseleit solution oxygenated (pH 7.4) (composition in mM: Nacl 95, KCl 5, MgSO_4_ 1.2, KH_2_PO_4_ 1.2, NaHCO_3_ 24.9, CaC_l2_ 2.6, glucose 10) and bubbled with a mixture of 95% O_2_ and 5% CO_2_. The surrounding connective tissue was carefully removed and the vessel was cut into 3 mm long rings, with special care taken to avoid damaging the endothelium. The rings of the proximal portion of the aortic arch were mounted horizontally under isometric conditions by inserting two stainless-steel wires into the lumen, according to the Bevan & Osher method (1972) [20]. Extreme care was taken not to stretch or damage the luminal surface of the aorta to ensure the integrity of the endothelium. The tissue isometric tension (g) was recorded by an isometric force transducer (F-60, Narco Biosystems, Inc., TX, USA) connected to a polygraph (MK III-S, Narco Biosystems, Inc., TX, USA). Tissues were allowed to equilibrate under an optimum final force of 1 g for a period of 60 min in a water-jacketed tissue bath (10 ml) containing oxygenated Krebs- Henseleit solution at 37°C, renewing the buffer every 20 min. After stabilization, the contractile function was verified by obtaining a KCl-induced contraction and the functional state of the endothelium was evaluated by the observation of relaxation to ACh on a ring precontracted with norepinephrine (NE). Only the endothelial intact rings (more than 50% relaxation to Ach) were used.

#### Aortic ring tension measurements

The vasoconstrictive responses were normalized to exposure to KCl (100 mmol/L) until a stable and repeatable level of constriction was obtained. This constriction value was recorded as the baseline of aortic rings constriction. After the washout (3 times for 20 min), the concentration response was assessed by adding cumulative concentrations of NE (1×10^−9^ to 1×10^−5^). The contractile response of the thoracic aortic rings to NE was defined as a percentage of the baseline. The concentration of NE producing the half-maximal contractile effect (i.e., EC_50_) and the maximal contractile effect were estimated by linear regression analysis (fitted to the Hill equation) from log concentration-response curves and expressed as −log EC_50_ (pD2 values).

After the washout (3 times for 20 min) and a period of recovery, cumulative concentration-response curves to sodium nitroprusside (SNP) were performed by adding cumulative concentrations (1×10^−9^ to 1×10^−5^) to the rat aortic rings submaximally contracted with NE. Vasodilation induced by SNP is expressed as a percentage of inhibition of NE-induced contraction. The concentration of SNP producing half-maximal relaxation (i.e., EC_50_) and the maximal relaxation of the NE contractile effect were estimated by linear regression analysis (fitted to the Hill equation) from log concentration-response curves and expressed as −log EC_50_ (pD2 values) and as percent of maximal relaxation.

### Transmission electron microscopy

Fifty transverse serial sections of the aortic arch were cut longitudinally, placed in 3% glutaraldehyde/1.5% paraformaldehyde solution buffered at pH 7.2 with 0.1 mol/l phosphate-buffered saline, and refrigerated at 4°C until processing. Samples were prepared for transmission electron microscopy by standard methodology. Briefly, samples were post fixed at 4°C for 1.5 h in 1% osmium tetroxide/1.5% potassium ferrocyanide solution. They were then washed in 0.1 mol/l phosphate-buffered saline, dehydrated in graded concentrations of ethanol, and embedded in Epon 618. The epoxy blocks were sliced on a LKB-V ultratome at a 70 to 80 nm thickness, stained with uranylacetate and lead citrate, and examined with a Hitachi Hu-12A transmission electron microscope. High-resolution digital images were acquired directly on a computer.

### Histological analysis

Specimens from the thoracic aorta were cut into 0.5-cm sections, fixed in 10% buffered formalin for 24 h, removed, and embedded in paraffin. The radial thickness of the media was measured on hematoxylin and eosin (H&E)-stained arterial sections using image analysis software (Image Pro Plus, version 4.5; Media Cybernetics, Silver Spring, MD, USA). Four measures per image were obtained at 0°, 90°, 180°, and 270° to determine media thickness.

### Immunofluorescence staining

The series of transverse cryostat sections of aortic tissue (10 µm) were fixed, opened longitudinally, and labeled using a polyclonal rabbit anti-Cx37 (1∶200), anti-Cx40 (1∶400), anti-Cx43 (1∶400) or anti-Cx45 (1∶100, Abcam, Cambridge, MA, USA), respectively. Subsequently, the sections were rinsed with PBS, followed by application of the secondary antibody goat anti rabbit IgG conjugated with CY-5 (1∶400, Life Technologies/Invitrogen, Carlsbad, CA). The primary antibody was omitted in the negative controls. Sections were then incubated for 30 minutes with 1 µg/ml DAPI, washed three times with PBS, mounted into Vectashield mounting medium (Baria, Germany) and viewed by confocal laser scanning microscopy using a Leica TCS SP equipped with an argon/krypton laser with the appropriate filter spectra adjusted for the detection of CY5 fluorescence.

### Western blot analysis

The thoracic aorta samples were processed as reported [9]. As primary antibodies, anti-Cx37 (1∶200, Santa Cruz Biotechnology, Inc, USA), anti-Cx40 (1∶400, Santa Cruz Biotechnology, Inc, USA), anti-Cx43 (1∶1000, Abcam, Cambridge, MA, USA), anti-Cx45 (1∶200, Abcam, Cambridge, MA, USA), anti NF-κB (p65) (1∶1000, Santa Cruz Biotechnology, Inc, USA), anti IκBα (1∶1000, Cell Signaling Technology, Inc, USA), anti IκBβ (1∶2000, Cell Signaling Technology, Inc, USA), and anti Phospho IκBα(Ser32) (1∶1000, Cell Signaling Technology, Inc, USA) antibody were used.

### RNA preparation and Real-time reverse transcription-PCR

RNA was analyzed by real-time PCR as described previously [11]. The PCR primers used were designed by Premier 5.0 (PREMIER Biosoft International, Palo Alto, CA, USA) based on published nucleotide sequences for rat Cx37 (forward: 5′- CAT CCG ATA CTG GGT GCT GC -3′; reverse: 5′-CGC CGA GAC AGG TAA ATG ACG-3′), rat Cx43 (forward: 5′-GAC ATG GGT GAC TGG AGT G-3′; reverse: 5′-TTG AGT GTT ACA GCG AAA GG-3′), rat Cx40 (forward: 5′-GAA AGA GGT GAA CGG GAA GA-3′; reverse: 5′-GCC ACA GCC ATC ATA AAG ACA-3), rat Cx45 (forward: 5′- GAG ATG GAG TTA GAA AGC GAG AA-3′; reverse: 5′- CAG GAA ATA CTG CCC TAT GAG A-3) and rat β-actin (forward: 5′- ACG GTC AGG TCA TCA CTA TCG-3′; reverse: 5′- GGC ATA GAG GTC TTT ACG GAT G-3).

### Statistical analysis

The data are expressed as the mean ± SD. The statistical significance of differences between the group means was determined by a one-way ANOVA. Concentration–response curves were compared by a repeated measurement ANOVA (SPSS 13.0, Chicago, IL, USA). P<0.05 was considered significant.

## Results

### Contraction study in the aorta

**Figure 1:**
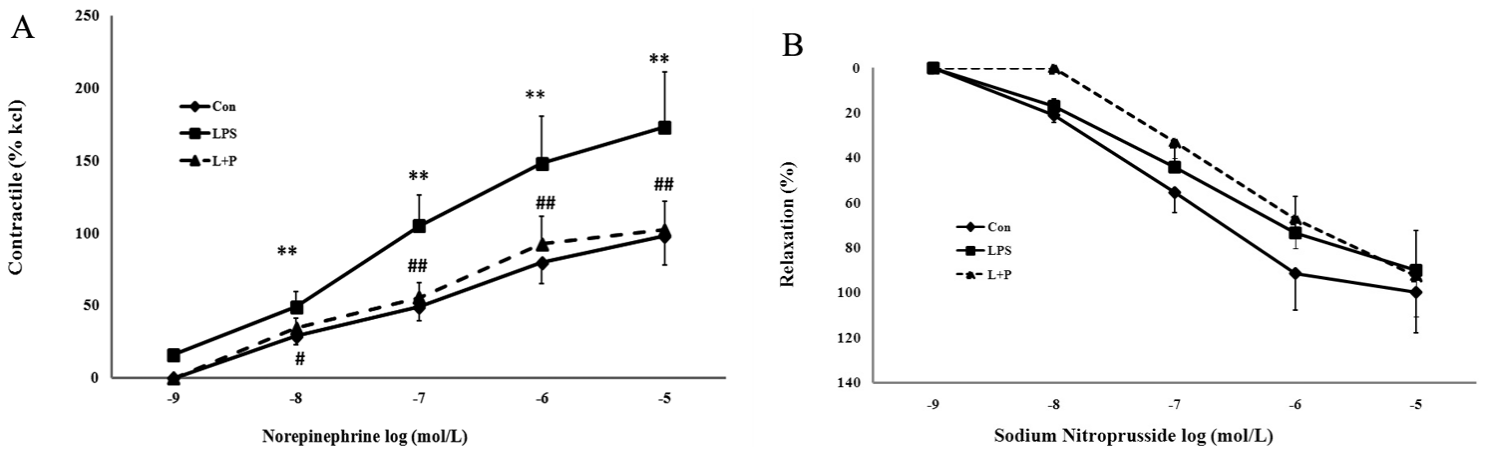
Contraction study (A) and relaxation study (B) in the aorta. The contractile responses are reported as a percentage of the maximum response to KCl. The relaxation responses to SNP are reported as the percentage of relaxation of the maximum contractile response. The data are presented as the mean ± SD. n = 6 in each group. **P<0.01 versus control, ^#^ *P*<0.05, ^##^ *P*<0.01 versus LPS group (a repeated measurement ANOVA). control group, offspring rats from maternal saline treatment; LPS group, offspring rats from maternal LPS exposure; L+P group, offspring rats from maternal LPS exposure+PDTC treatment.

NE produced a concentration-dependent contraction in the aortic rings. Compared to the offspring in the control group, contractile responses to NE in the aortic rings isolated from the offspring in the prenatal LPS exposure group were significantly increased for concentrations from 10^−8^ mol/L to 10^−5^ mol/L (*P*<0.01). The maximum contractions of the aortic rings were also obviously increased in the LPS exposure offspring group (*P*<0.01). In the maternal LPS exposure plus PDTC treatment group, the contractile responses of aortic rings to NE, from 10^−8^ mol/L to 10^−5^ mol/L, obviously decreased in offspring rats compared with offspring from maternal LPS exposure (*P*<0.05 or 0.01). Notably, the maximal response of NE contraction in the aortic rings isolated from the prenatal LPS exposure offspring showed a trend towards lesser relaxation at simply 10^−5^ mol/L NE. On the contrary, offspring in the maternal LPS exposure plus PDTC treatment group showed the trend towards lesser relaxation at all other NE concentrations. In additon, sensitivity to NE, expressed by the pD2 values, was not significantly different between experimental groups. (Figure 1A, Table 1). Meanwhile, no significant differences between female and male offspring rats were detected.

**Table 1:** Summary of pD2 and Maximal Response Values (%, n = 6, 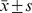).

		Con	LPS	L+P
NE	Maximal Response	96.88±20.22	170.38±35.17**	110.42±24.36#
	pD2	7.09±1.48	7.37±1.39	6.94±1.34
SNP	Maximal Response	100±0	81.73±20.37	88.75±22.34
	pD2	7.12±1.52	6.87±1.39	6.81±2.13

pD2 indicates −log EC50; NE, norepinephrine; SNP, sodium nitroprusside.

Maximal response of NE and SNP were calculated by linear regression analysis.

**P<0.01 versus control, ^#^P<0.05 versus LPS group (One-way ANOVA). control group, offspring rats from maternal saline treatment; LPS group, offspring rats from maternal LPS exposure; L+P group, offspring rats from maternal LPS exposure+PDTC treatment.

### Relaxation study in the aorta

SNP caused a concentration-dependent relaxation in the precontracted aortic rings. Although significance was not achieved, aortic rings isolated from the offspring in the prenatal LPS exposure group presented a lesser relaxation and attenuated maximal response to SNP compared with control offspring rats. In addition, the difference of aortic relaxation was not detected between offspring rats from maternal LPS exposure plus PDTC treatment and from maternal single LPS exposure. (Figure 1B, Table 1). Likewise, there were no gender differences.

### Morphological changes in the aorta

**Figure 2:**
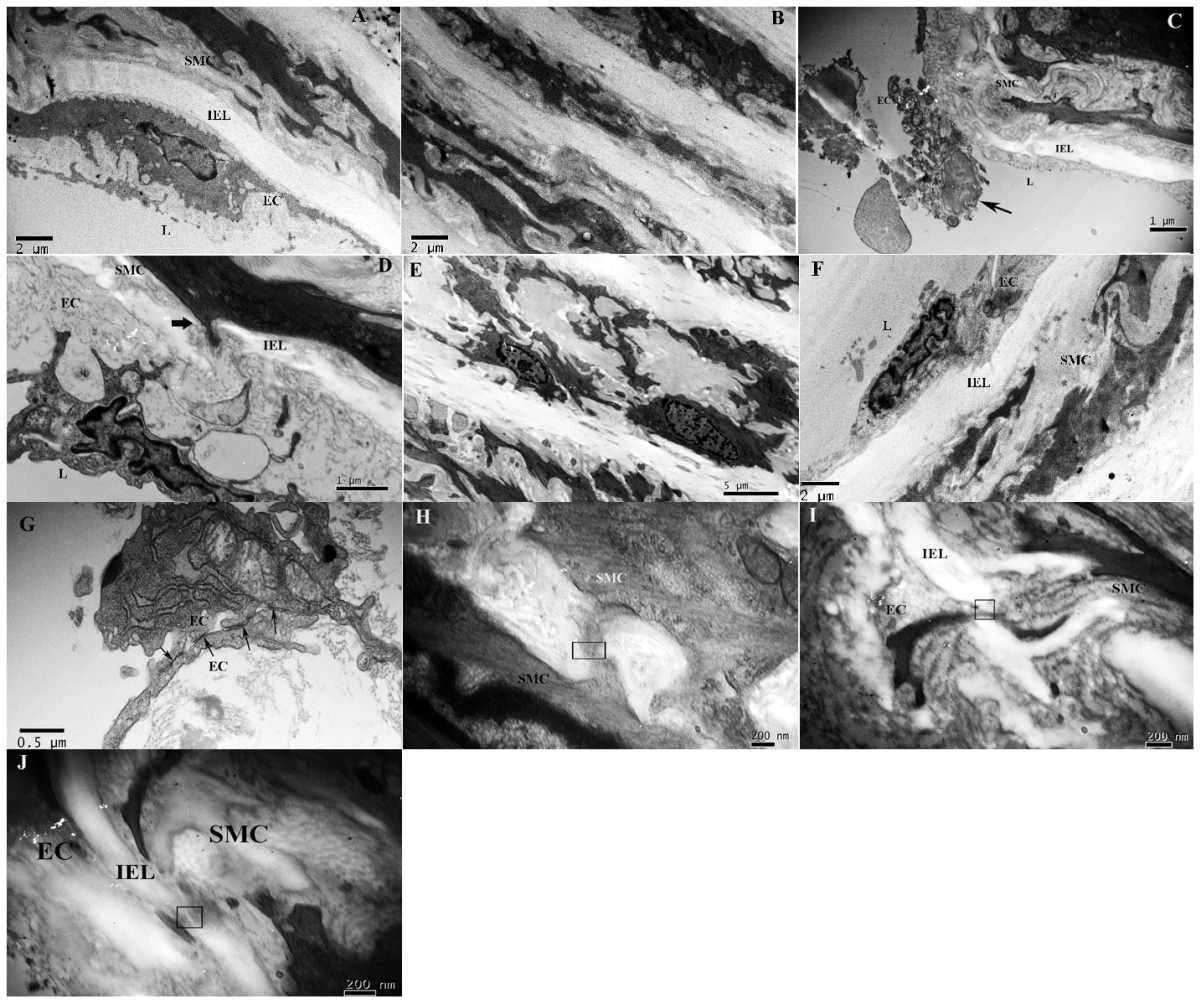
Representative transmission electron micrographs of aortas. A and B, control group. C–E, LPS group; C: desquamated ECs (arrowhead); D: SMCs migration (arrowhead). F, L+P group. G: gap junction between ECs (arrowhead) in LPS group. H: gap junctions between SMCs (box,) in LPS group. I: myoendothelial gap junction (box) in LPS group. J: myoendothelial association (box) in LPS group.

Using electron microscopy, no detectable pathological changes in the aorta were observed in the control offspring without maternal LPS exposure. All of the three layers in the vessel wall were intact, clear, and regularly arrayed and parallel (Figure 2A and B). However, the thoracic aorta of the maternal LPS exposure offspring exhibited lesions, and some of the endothelial cells were seen to be edematous, necrotic, detached from the basement membrane, or even desquamated. Local platelet adhesion and aggregation and microthrombosis were occasionally observed in the surface of the aortic lumen. Edema and thickening of the subendothelial layer and fragmentation of the internal elastic lamina were also observed. SMCs were often observed migrating from the media into the intima and breaking through the internal elastic lamina. Proliferation of the tunica media was also seen, in which disarranged SMCs with migration trends, mitochondrial swelling in some of the SMCs, proliferation of collagen fibers, and unclear elastic fiber layers were exhibited (Figure 2C, D and E). Additionally, the above pathological appearances were even less prominent when it was complicated with prenatal PDTC treatment (Figure 2F).

Maternal LPS exposure-induced aortic subcellular changes in the offspring rats were accompanied by cell-to-cell junction alterations. The long sections of well-defined gap junctions between ECs in the endothelial layer and small gap junctions between the SMCs were observed in the thoracic aortas from the control and prenatal LPS exposure offspring rats (Figure 2G, H), although there were significantly fewer gap junctions in the prenatal LPS exposure offspring rats. In addition, prenatal PDTC treatment did not have obvious effects on the loss of gap junctions that resulted from prenatal LPS exposure. The main criterion for identification of myoendothelial gap junctions (MEGJs) was the presence of the characteristic pentalaminar membrane structure at points of cell-cell contact, wherever the central region had higher electron opacity than the inner parts. Electron microscopic examination revealed that ECs and SMCs were separated by the relatively large internal elastic lamina in the thoracic aorta, and ECs sent protrusions toward SMCs, the majority of which were rather short, resulting in rarely observed MEGJs in the three groups (Figure 2I). Actually, it was feasible that frequently observed contacts between two types of cells within the vascular wall of the thoracic aorta should be referred to as myoendothelial associations rather than as MEGJs (Figure 2J). There were no significant differences between female and male offspring rats.

### The medial thickness of the aorta

**Figure 3:**
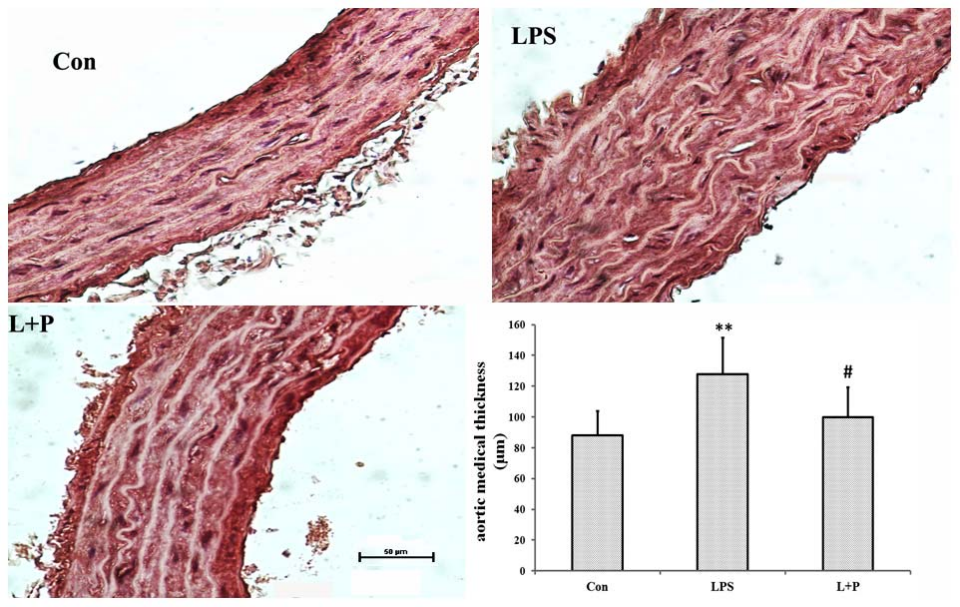
The medial thickness of the aortas. Histopathologic observation of the aortic medial thickness (H&E staining); Morphometric analysis of the aortic medial thickness. The data are presented as the mean ± SD. n = 6 in each group. **P<0.01 versus control, ^#^ *P*<0.05 versus LPS group (One-way ANOVA). control group, offspring rats from maternal saline treatment; LPS group, offspring rats from maternal LPS exposure; L+P group, offspring rats from maternal LPS exposure+PDTC treatment.

Aortic HE staining revealed markedly aortic medial growth from the prenatal LPS exposure offspring rats compared with normal offspring rats without maternal LPS exposure (*P*<0.01). However, PDTC treatment added to maternal LPS exposure obviously prevented the increase in medial radial thickness (*P*<0.05) (Figure 3).

### Immunolabeling of Cx37, Cx40, Cx43 and Cx45

**Figure 4:**
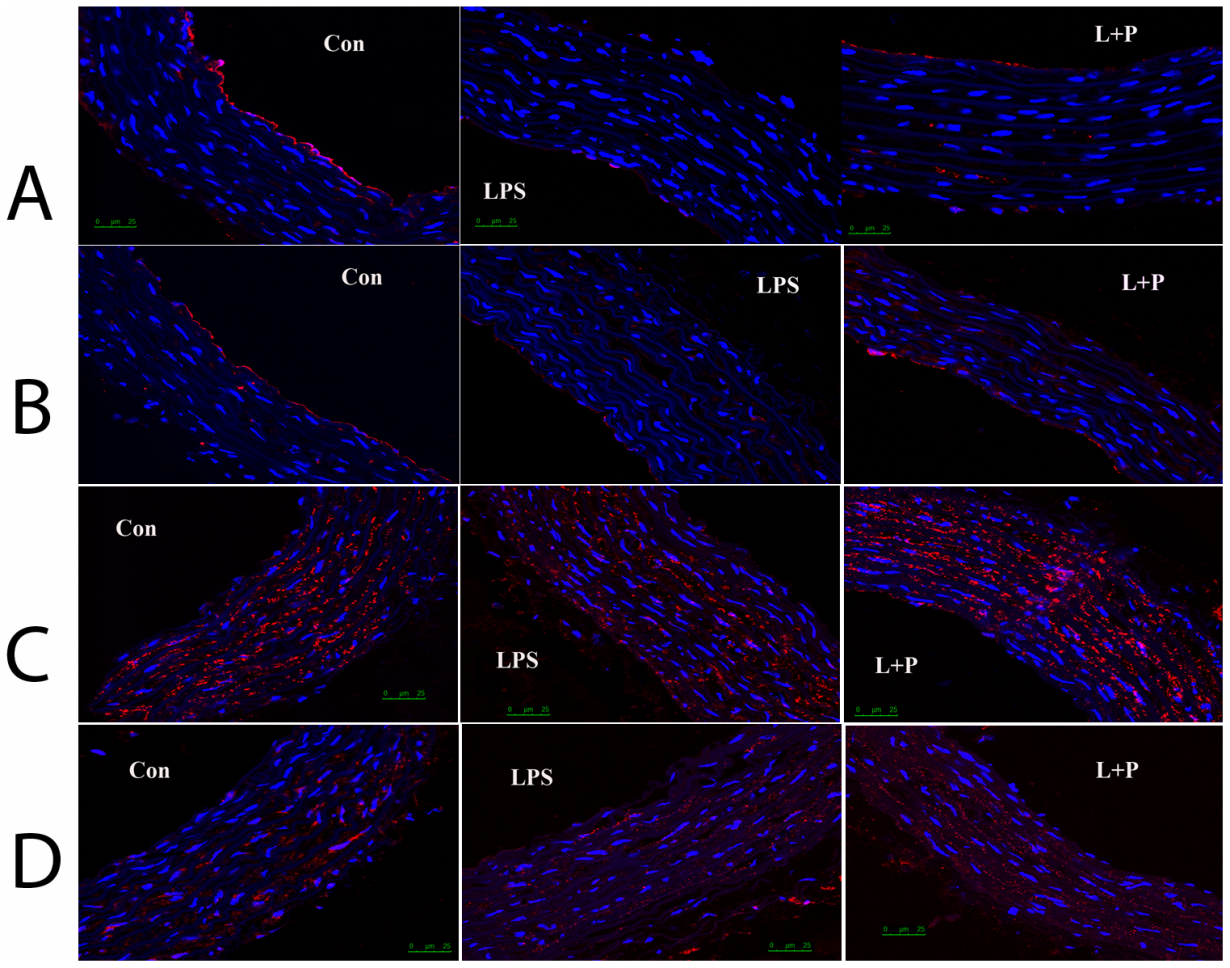
Immunolabeling of Cx37 (A), Cx40 (B), Cx43(C) and Cx45 (D) in the aorta. control group, offspring rats from maternal saline treatment; LPS group, offspring rats from maternal LPS exposure; L+P group, offspring rats from maternal LPS exposure+PDTC treatment.

Immunofluorescent patterns of Cx37 and Cx40 were exhibited predominantly in the endothelium of the aorta. However, the fluorescence of Cx43 and Cx45 were both expressed abundantly in the endothelial and smooth muscle cells of the aorta in all experimental groups. Furthermore, a local marked decrease in the number and intensity of Cx37 fluorescent spots was observed in the endothelium of the maternal LPS exposure offspring compared to the control offspring without maternal LPS exposure. In contrast, PDTC prenatal treatment significantly attenuated the decrease in the number and intensity of Cx37 fluorescent spots resulting from maternal LPS exposure. Notably, no differences in the number and pattern of Cx40, Cx43 and Cx45 spots were detected in the control offspring, the maternal LPS exposure offspring or the maternal LPS exposure plus PDTC treatment offspring (Figure 4).

### Expressions of Cx37, Cx40, Cx43 and Cx45

**Figure 5:**
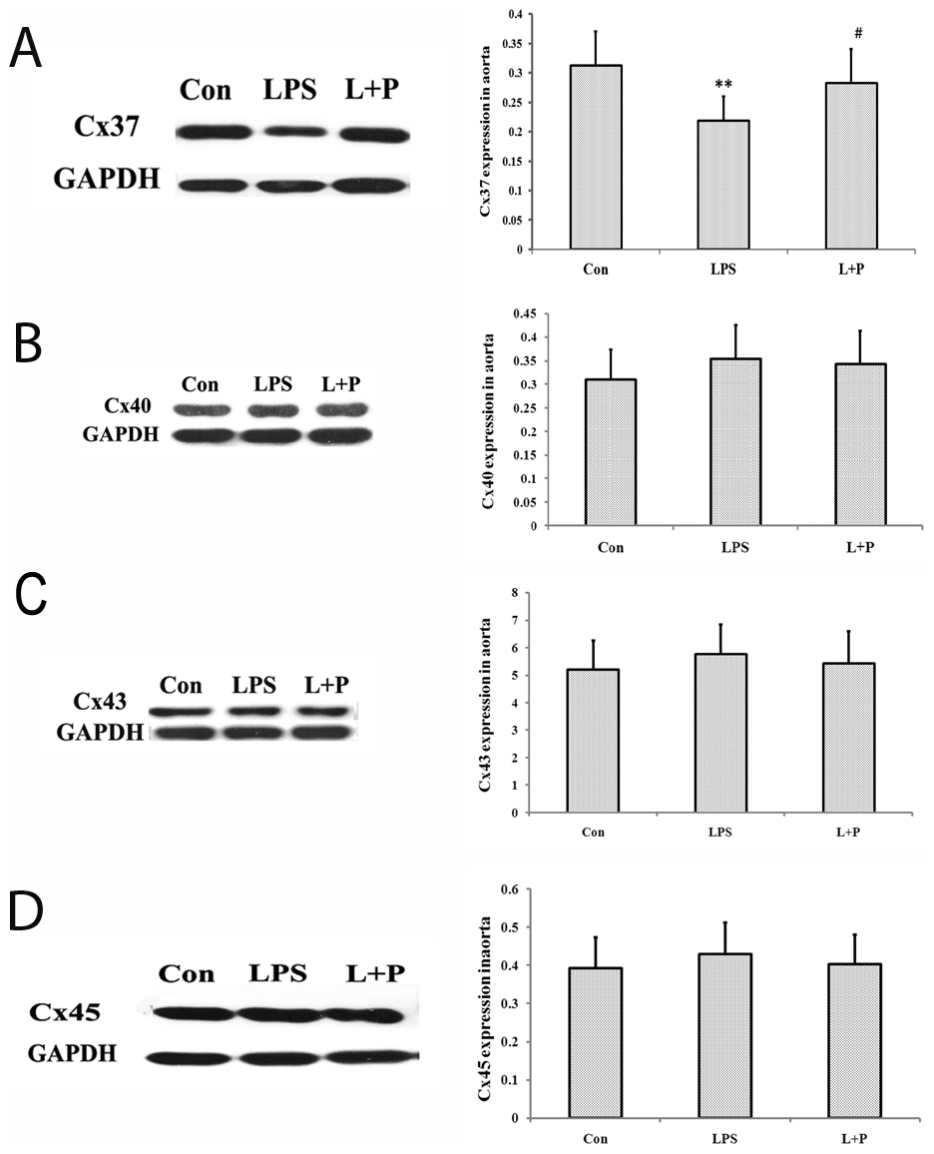
Expressions of Cx37 (A), Cx40 (B), Cx43 (C) and Cx45 (D) in the aorta. The data are presented as the mean ± SD. n = 7 in each group. **P<0.01 versus control, ^#^ *P*<0.05 versus LPS group (One-way ANOVA). control group, offspring rats from maternal saline treatment; LPS group, offspring rats from maternal LPS exposure; L+P group, offspring rats from maternal LPS exposure+PDTC treatment.

Accordingly, western blot analysis of the aorta homogenates showed that the expression of Cx37 (*P*<0.01), but not Cx40, Cx43, or Cx45 was significantly decreased in the maternal LPS exposure offspring group compared with the control offspring group. However, offspring rats from the maternal LPS exposure plus PDTC treatment showed an increase in the expression of Cx37 compared to that from single maternal LPS exposure (*P*<0.05) (Figure 5).

### Messengers RNA expressions of Cx37, Cx40, Cx43 and Cx45

**Figure 6:**
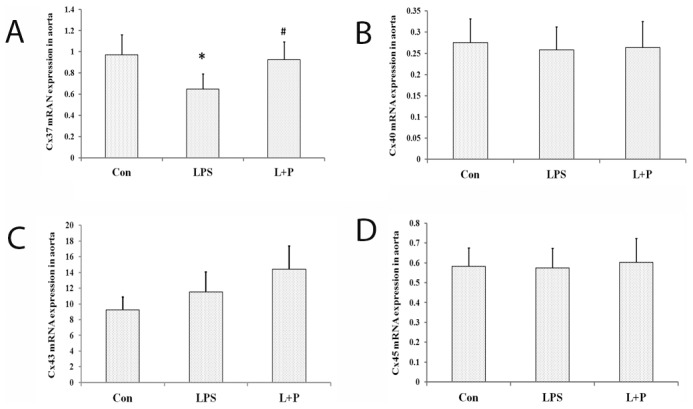
Messenger RNA expressions of Cx37 (A), Cx40 (B), Cx43(C) and Cx45 (D) in the aorta. The data are presented as the mean ± SD. n = 7 in each group. *P<0.05 versus control, ^#^ *P*<0.05 versus LPS group (One-way ANOVA). control group, offspring rats from maternal saline treatment; LPS group, offspring rats from maternal LPS exposure; L+P group, offspring rats from maternal LPS exposure+PDTC treatment.

Compared to the control offspring rats, the Cx37 mRNA expression was significantly decreased in the maternal LPS exposure offspring (*P*<0.05). The decreased mRNA expression of Cx37 in the maternal LPS exposure offspring was obviously reversed when it was complicated with PDTC treatment (*P*<0.05). The mRNA expression of Cx40, Cx43 and Cx45 in the aortas, however, did not differ significantly among the different treatment offspring rats (Figure 6).

### Expressions of p65 NF-κB, IκBα, phospho-IκBα and IκBβ

**Figure 7:**
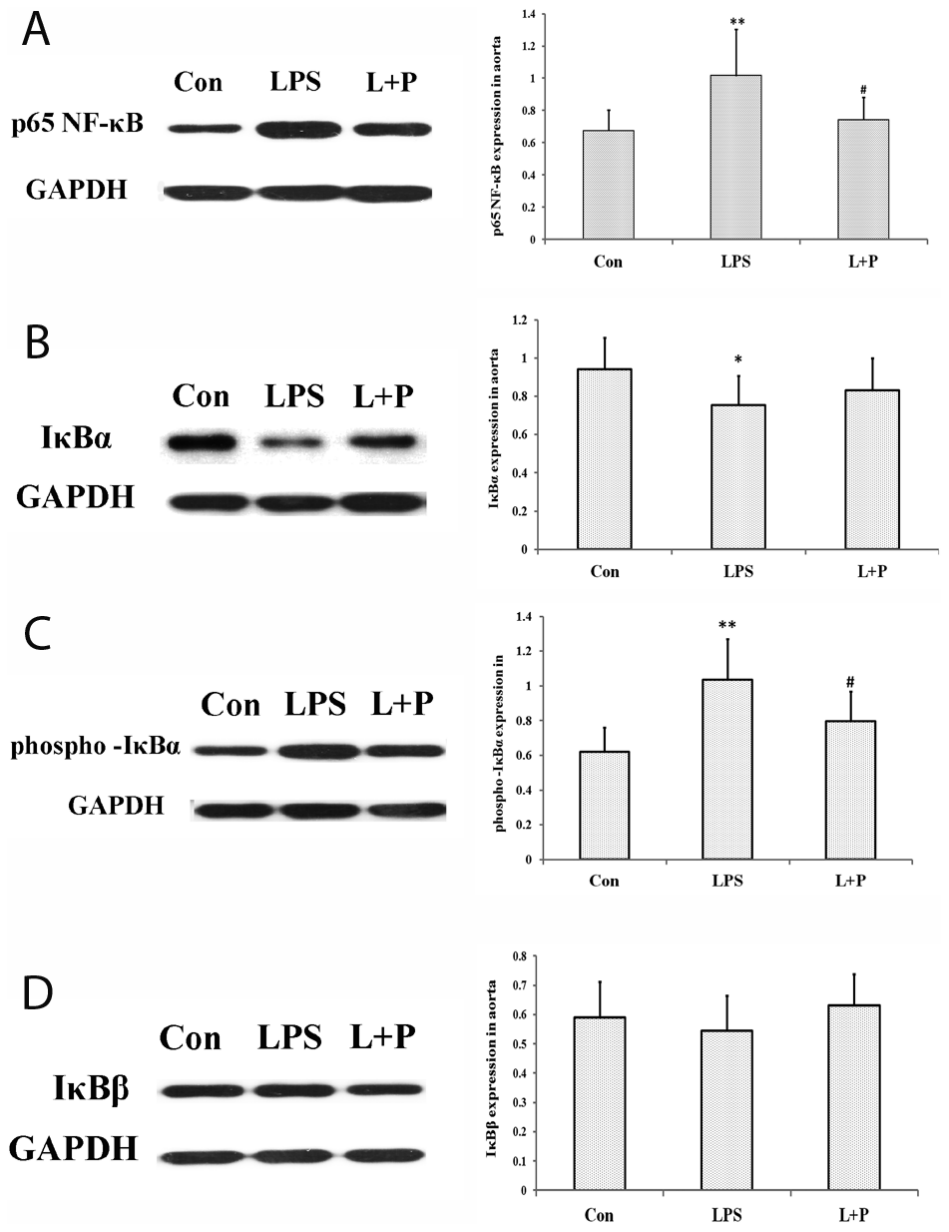
Expressions of p65 NF-κB (A), IκBα (B), phospho-IκBα (C) and IκBβ (D) in the aorta. The data are presented as the mean ± SD. n = 7 in each group. *P<0.05, **P<0.01 versus control, ^#^ *P*<0.05 versus LPS group (One-way ANOVA). control group, offspring rats from maternal saline treatment; LPS group, offspring rats from maternal LPS exposure; L+P group, offspring rats from maternal LPS exposure+PDTC treatment.

Finally, western blot analysis of the aorta homogenates demonstrated the significantly increased expression of NF-κB (p65) (*P*<0.01) and phospho-IκBα (*P*<0.01), whereas a slight decrease in the expression of IκBα (*P*<0.05) was also detected in maternal LPS exposure offspring compared with control offspring rats. Inhibition of NF-κB (p65) (*P*<0.05) activation by maternal PDTC treatment produced obviously decreased expression of phospho-IκBα (*P*<0.05), but no obvious effects on IκBα expression. There was no significant difference in the expression of IκBβ in the aorta between each of the treatment offspring rats (Figure 7).

## Discussion

There is accumulating recognition that hostile perinatal environments influence fetal organogenesis and contribute to adult diseases [4]–[6]. Specifically, maternal inflammation is common during pregnancy and has adverse repercussions on the developing fetus [21]. In rodent models, low-dose systemic LPS administration could induce inflammatory responses in the pregnant dam, and subsequently, maternally derived inflammatory mediators rapidly cross the placenta and are transmitted to the fetus [22], [23]. Our microarray analyses have indicated alterations in the gene expression of the developmental and metabolic status in embryos obtained from LPS-injected dams, which appear to be genetically predisposed to diseases later in life [24].

Recently, decreased aortic compliance has been regarded as an independent risk factor for cardiovascular-related complications [25], [26]. Endothelial cells underlie the key components of arterial contraction and relaxation and are thus essential for the control of vessel tone, blood flow and pressure. In the current study, prenatal LPS exposure exhibited impairment of endothelial cells in the aorta. An intimal thickening response to the endothelial injury involved broken elastic fiber layers, migration and proliferation of SMCs. Meanwhile, impaired vascular reactivity in the maternal LPS exposure offspring was detected, which was characterized by an increased contraction in response to agonists in the aortic rings, however, the relaxation evoked by Ach or SNP was unchanged. This study provided the first evidence that systemic maternal inflammation exposure produces vascular impairment of the aorta in 12-wk-old offspring rats. Furthermore, these abnormal changes were non-gender specific. In addition, morphological abnormalities in aorta independently of blood pressure were observed obviously at the 1-day-old offspring rats [27], and accumulating of evidences have revealed the correlation between aortic impairments and the level of blood pressure [28], [29]. In this regard, prenatal LPS exposure leads to impairment of the aorta in 12-wk-old offspring rats, which possibly induced by prenatal LPS exposure, however, elevated blood pressure could not been rule out.

Gap junctions are intercellular channels that directly connect the cytoplasm of adjacent cells, allowing for the passage of currents and small signaling molecules. The aorta, which is a sparsely innervated and electrically quiescent vessel, is likely to be particularly dependent on gap junctional communications for coordinating the response of endothelial cells and SMCs to diverse signals [30]. The properties of gap junctional channels are determined by the connexins, proteins that belong to a multigene family. Cx37, together with Cx40, Cx43 and Cx45, represent major gap junction proteins expressed in the large vessels including the aorta [31]. Gap junctions were implicated in the regulation of vascular tone as well as in the control of vascular cell proliferation and migration [30], [32]. In this regard, gap junctions may play an important role in the development of the vasculature and in responses to blood vessel damage [33]. To date, several studies have revealed that hypertension alters connexins expression in the vascular system [34]–[36]. However, there is little information on the pre-hypertensive period. Our current study demonstrated ultrastructural losses of gap junction, alterations of Cx37 distribution in the endothelial monolayer, and decreased Cx37 expression in the aortic walls of maternal LPS exposure offspring at 12 weeks of age, which was characterized by elevated blood pressure but not by hypertension according to the previous study. In contrast, maternal LPS exposure had no effect on the distribution and expression of Cx40, Cx43 and Cx45 in offspring. The results implied that attenuated gap junction maybe due to the decreased expression and function of Cx37 in the aortic wall of maternal LPS exposure offspring. Inhibition of gap junction communication in the vascular wall has been reported to result in contractional dysfunction through endothelium dependent mechanisms [37], [38]. In keeping with this finding, the role of Cx37 in the vasculature has previously been analyzed in mice deficient for Cx37, which showed impaired conduction of arteriolar vasoconstriction [39]. Moreover, Cx37 expression is decreased in response to factors inducing endothelial dysfunction [40], [41]. Therefore, these observations documented that alterations in Cx37 expression may contribute to the occurrence of vascular dysfunction demonstrated in the 12-wk-old offspring of prenatal LPS exposure.

In addition, it should be noted that the expression of Cx43 was increased in the aortic wall of the 16-wk-old maternal LPS exposure offspring, accompanying a further elevation in blood pressure (data not shown). Knock-in studies have suggested that connexins may have shared and unique functions [42]. In our study, the expression patterns of gap junctions and connexins changed in the offspring of prenatal LPS exposure, and interestingly, the alterations were inconsistent during the course of increased blood pressure, which implied that adequate function may be maintained irrespective of connexin type. Accordingly, we speculated that alterations in the patterns of connexin expressions represent an adaptive response of offspring rats to the prenatal LPS exposure occurring in the vascular wall during hypertension, possibly to maintain vascular homeostasis.

In the present study, maternal LPS exposure offspring rats exhibited chronic vascular activation of nuclear factor (NF)-κB, characterized by a decrease in IκBα expression, an increase in phospho-IκBα and p65 NF-κB expression. Activation of NF-κB is regarded to be an important step in the development of vascular damage [43], [44], and appears to be a promising molecular target for the treatment of inflammatory vascular diseases [45], [46]. We have reported that the NF-κB inhibitor, PDTC, which inhibited the renal activation of NF-κB induced by prenatal exposure to LPS, lead to a nearly complete reversal in renal function and a partial reverse in blood pressure [47]. Our current data also indicated that offspring rats from maternal LPS exposure plus PDTC treatment exhibited attenuated vascular activation of NF-κB along with improved vascular impairment and functional dysfunction, including abnormal vasoconstriction. Meanwhile, maternal LPS exposure plus PDTC treatment restored the expression patterns of gap junctions and connexins in offspring rats, with an increase in Cx37 expression included. Supporting evidence for the importance of Cx37 coupling in NO synthase (eNOS) activity has been provided. It is conceivable that in vivo, the inhibitory property of Cx37 on endothelial eNOS may mostly affect reactive oxygen species (ROS) production under pathological conditions rather than NO synthesis in more physiological states [48]. We have recently demonstrated that prenatal LPS exposure induced the local increased expression of eNOS, Ang II, whereas it included a decrease in eNOS phosphorylation in the aorta of offspring rats [10]. It has been shown that Ang II induces target organ damage by activating the NF-κB signaling pathway, and many signals that lead to activation of NF-κB converge on the ROS-dependent activation of a high-molecular-weight complex that contains an IκB kinase [49], [50]. Therefore, taken together these findings suggest that Cx37 mediates vascular dysfunction, at least in part, through modulation of local NF-κB activation in aorta.

In conclusion, these results elucidated that maternal LPS exposure during pregnancy lead to offspring vascular dysfunction at 12 weeks of age, which appear to be genetically predisposed to cardiovascular disease later in life. Alterations in patterns of connexin expressions, particularly decreased expression of Cx37, contributed to the aortic impairment, which may be associated with NF-κB activation. Measures taken to prevent maternal inflammatory responses and other adverse maternal challenges would likely be useful in the prevention of cardiovascular diseases in adult offsprings.
